# The Psychological Impact of COVID-19 Pandemic on Suicidal Thoughts in the United Kingdom

**DOI:** 10.1192/bjo.2022.238

**Published:** 2022-06-20

**Authors:** Shanaya Rathod, Peter Phiri, Saseendran Pallikadavath, Elizabeth Graves, Ashlea Brooks, Pranay Rathod, Sharon Lin

**Affiliations:** 1 Southern Health NHS Foundation Trust, Southampton, United Kingdom; 2 University of Porstmouth, Portsmouth, United Kingdom; 3 London, United Kingdom; 4 University of Southampton, Southampton, United Kingdom

## Abstract

**Aims:**

Background: The impact of the pandemic and resultant restrictions on suicidal thoughts may vary across populations, geographical areas, between high and low socio-economic groups and vulnerable populations. Aim: To investigate the psychological impact of COVID-19 and resultant restrictions on suicidal thoughts in the United Kingdom.

**Methods:**

The study group conducted a cross sectional survey using a questionnaire based on published approaches (Generalised Anxiety Disorder 7, Patient Health Questionnaire 9, Impact of Events Scale-Revised) to understand the psychological impact of COVID-19 and the resultant restrictions on suicidal thoughts. The study was conducted in 3 phases to capture the different phases of the pandemic restrictions:

- Phase 1: 1st May 2020 to 31st July 2020
- Phase 2: 12th November 2020 to 12th February 2021
- Phase 3: 1st July 2021 to 30th September 2021

Inclusion: All individuals above 16 years of age who wanted to participate were eligible.

Analysis strategy: Descriptive analysis and logistic regression is applied in this study.

**Results:**

The study recruited 29133 participants in phase 1; 83851 participants in phase 2 and 75204 participants in phase 3. The largest age group of participants was 45–64 years. About two thirds of respondents were female. Majority of participants were of White British ethnicity. 31% participants in phase 1, 30% in phase 2 and 19% in phase 3 reported suicidal thoughts.

The preliminary regression analysis indicates that younger and male participants reported more suicidal thoughts among other findings which will be reported in the presentation.

Limitations: The non-probability sample design and time limited surveys meant that longitudinal changes were not possible to elicit.

**Conclusion:**

There is mixed evidence on whether rates of suicidal thoughts increased during the pandemic. The results of this study will add to the evidence base and influence future pandemic planning and efforts to developing resilience and good mental health in society.

